# Evaluation of indication in a urinalysis driven reflex urine culture protocol at an academic medical center

**DOI:** 10.1017/ash.2023.306

**Published:** 2023-09-29

**Authors:** Mackenzie Keintz, Jasmine Marcelin, Mark Rupp, Trevor Van Schooneveld

## Abstract

**Background:** Asymptomatic bacteriuria (ASB) is a widespread problem in hospitalized patients in which only a small subset of patients benefit from treatment. Other patient populations with ASB are harmed by treatment. In 2014, our institution implemented a urinalysis (UA)-driven reflex culture protocol which evaluated patient symptoms, risk factors, and the UA to determine whether bacterial culture was performed (Fig. 1). The goal of this process was to ensure that urine cultures were only performed in those patients who had symptoms of UTI and an abnormal urinalysis while allowing for exceptions in populations where treatment of ASB may be appropriate (ie, pregnancy, aged <3 years, impending urologic surgery, kidney transplant) or where the urinalysis may not be useful in determining whether infection is present (ie, neutropenia). An “other” indication with free-text documentation required was included to allow for unique situations. We evaluated the free-text option to determine whether additional indications were needed and whether data entered were medically appropriate. **Methods:** This retrospective review at a Midwestern, tertiary-care, academic medical center included inpatient UA with UTI evaluation order sets between July 1, 2020, and June 30, 2022. Descriptive statistics analyzed order-set utilization. **Results:** In total, 35,469 “urinalysis to reflex culture” order sets were submitted, of which 9,493 resulted in culture. Of these, 839 (8.8%) were ordered with an indication of “other.” “Other” was the most cited indication for special population override contributing to 40% (n = 839 of 2,085) of these indications, followed by kidney or pancreas transplant (29%) and neutropenia (13%). The write-in options fell into 1 of 11 themes (Fig. 2). The 3 most common reasons a urine culture was obtained using the free-text option were nonurologic surgical intervention (n = 223 of 839), immunosuppression not otherwise defined (n = 195 of 839), and symptom presence (n = 146 of 839). Based on current literature, 97% of other indications were inappropriate (n = 816 of 839). If the UTI protocol had been strictly followed, 696 of 839 (83%) cultures ordered with an indication of “other” would not have been obtained, due either to lack of symptoms or, if symptomatic, lack of pyuria. **Conclusions:** Most cultures obtained by selecting the “other” special population option on the algorithm were obtained in situations in which a urine culture was unnecessary. Removing the “other” indication from the algorithm may improve appropriateness of urine culturing with a possible decrease in CA-UTI and treatment of ASB. Although most write in rationales were inappropriate, adding an additional category for deceased donor-organ evaluation would be reasonable.

**Figure:**
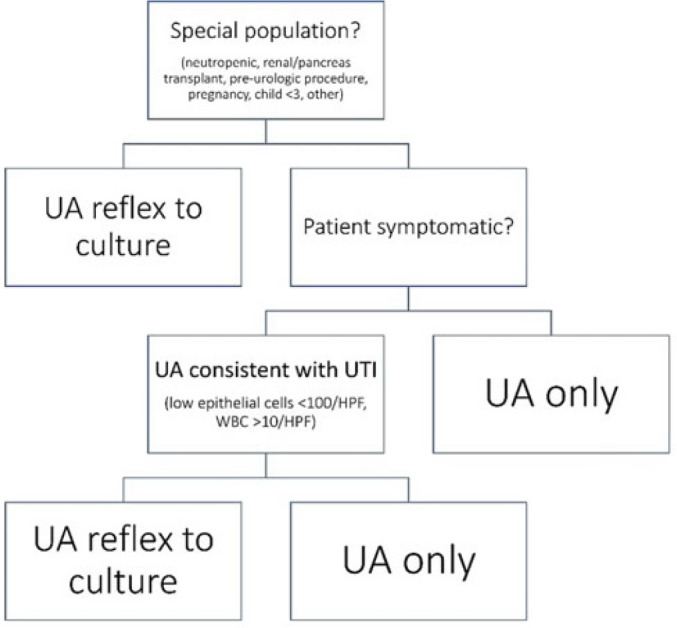

**Figure:**
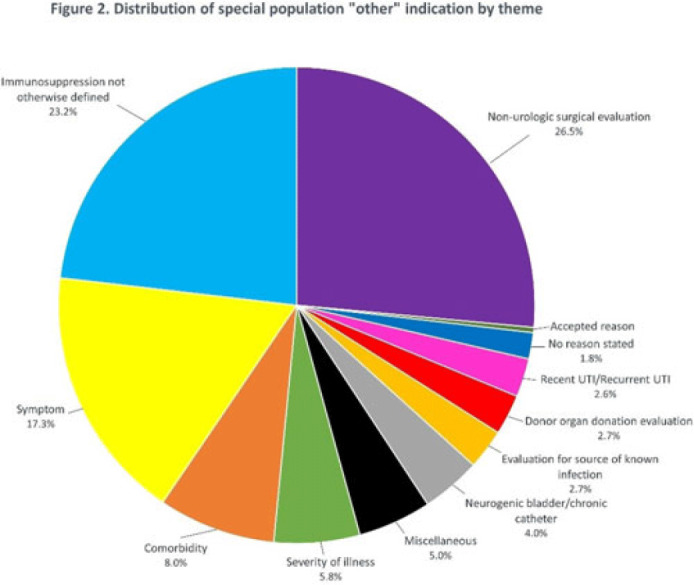

**Disclosures:** None

